# Theoretical Encapsulation of Fluorouracil (5-FU) Anti-Cancer Chemotherapy Drug into Carbon Nanotubes (CNT) and Boron Nitride Nanotubes (BNNT)

**DOI:** 10.3390/molecules26164920

**Published:** 2021-08-13

**Authors:** Maryam Zarghami Dehaghani, Farrokh Yousefi, S. Mohammad Sajadi, Muhammad Tajammal Munir, Otman Abida, Sajjad Habibzadeh, Amin Hamed Mashhadzadeh, Navid Rabiee, Ebrahim Mostafavi, Mohammad Reza Saeb

**Affiliations:** 1 Center of Excellence in Electrochemistry, School of Chemistry, College of Science, University of Tehran, Tehran 11155-4563, Iran; maryam.zarghamid@gmail.com; 2 Department of Physics, University of Zanjan, Zanjan 45195-313, Iran; yousefi.farrokh@gmail.com; 3 Department of Nutrition, Cihan University-Erbil, Kurdistan Region, Erbil P.O. Box 625, Iraq; smohammad.sajadi@gmail.com; 4 Department of Phytochemistry, SRC, Soran University, Soran P.O. Box 624, Iraq; 5 College of Engineering and Technology, American University of the Middle East, Egaila 54200, Kuwait; muhammad.munir@aum.kw.edu (M.T.M.); otman.abida@aum.edu.kw (O.A.); 6 Department of Chemical Engineering, Amirkabir University of Technology (Tehran Polytechnic), Tehran 1591639675, Iran; 7 Mechanical and Aerospace Engineering, School of Engineering and Digital Sciences, Nazarbayev University, Nur-Sultan 010000, Kazakhstan; 8 Department of Physics, Sharif University of Technology, Tehran P.O. Box 11155-9161, Iran; nrabiee94@gmail.com; 9 Stanford Cardiovascular Institute, Stanford University School of Medicine, Stanford, CA 94305, USA; 10 Department of Medicine, Stanford University School of Medicine, Stanford, CA 94305, USA; 11 Department of Polymer Technology, Faculty of Chemistry, Gdańsk University of Technology, G. Narutowicza 11/12, 80-233 Gdańsk, Poland; mrsaeb2008@gmail.com

**Keywords:** drug delivery, carbon nanotubes, boron nitride nanotubes, chemotherapy, drug delivery system, molecular dynamics, DREIDING force field, anti-cancer drug, Fluorouracil

## Abstract

Introduction: Chemotherapy with anti-cancer drugs is considered the most common approach for killing cancer cells in the human body. However, some barriers such as toxicity and side effects would limit its usage. In this regard, nano-based drug delivery systems have emerged as cost-effective and efficient for sustained and targeted drug delivery. Nanotubes such as carbon nanotubes (CNT) and boron nitride nanotubes (BNNT) are promising nanocarriers that provide the cargo with a large inner volume for encapsulation. However, understanding the insertion process of the anti-cancer drugs into the nanotubes and demonstrating drug-nanotube interactions starts with theoretical analysis. Methods: First, interactions parameters of the atoms of 5-FU were quantified from the DREIDING force field. Second, the storage capacity of BNNT (8,8) was simulated to count the number of drugs 5-FU encapsulated inside the cavity of the nanotubes. In terms of the encapsulation process of the one drug 5-FU into nanotubes, it was clarified that the drug 5-FU was more rapidly adsorbed into the cavity of the BNNT compared with the CNT due to the higher van der Waals (vdW) interaction energy between the drug and the BNNT. Results: The obtained values of free energy confirmed that the encapsulation process of the drug inside the CNT and BNNT occurred spontaneously with the free energies of −14 and −25 kcal·mol^−1^, respectively. Discussion: However, the lower value of the free energy in the system containing the BNNT unraveled more stability of the encapsulated drug inside the cavity of the BNNT comparing the system having CNT. The encapsulation of Fluorouracil (5-FU) anti-cancer chemotherapy drug (commercial name: Adrucil^®^) into CNT (8,8) and BNNT (8,8) with the length of 20 Å in an aqueous solution was discussed herein applying molecular dynamics (MD) simulation.

## 1. Introduction

Cancer, as the second reason for human mortality, arises from irregular cell growth and has become one of the major issues in medical science [1,2]. According to the statistics provided by the International Agency for Research on Cancer, there were globally about 18.1 million new cases of cancer reported in 2018, with 9.6 million fatalities attributed to the cancer-related illnesses reported in the same year [3,4]. Nevertheless, there are serious complexities and challenges associated with understanding the mechanism of performance and side effects of chemotherapeutics. Henceforth, several therapeutic approaches have been developed to combat cancerous cells, individually or combined with surgery, radiation therapy, hyperthermia therapy and chemotherapy [5,6]. In terms of chemotherapy as a conventional cancer therapy, anti-cancer drugs used in chemotherapy can be classified into six main groups based on their action mechanism on cancer cells: (*i*) mitotic inhibitors which hinder cell mitosis, (*ii*) topoisomerase inhibitors that inhibit DNA topoisomerases during replication, (*iii*) alkylating agents which facilitate the DNA damage, (*iv*) anti-tumor antibiotics which are in all phases of the cell cycle and act through interfering enzymes involved in DNA replication, (*v*) anti-metabolites lead to the replacement of the building blocks of DNA and RNA and (*vi*) corticosteroids, which act as inflammatory agents in cancer treatment [7,8].

Despite the broad application of chemotherapy in cancer treatment, some possible drawbacks of anti-cancer drugs used in this therapeutic approach, such as toxic side effects on fast-growing healthy cells and multidrug resistance, would limit their further usage in cancer treatment [9,10]. In this regard, nanoscale delivery systems have been introduced to overcome support chemotherapy-assisted therapeutics by providing delaying drug resistance [11,12], sustained and targeted drug release into the tumor microenvironment [13,14], facilitating the penetration of the drug into the cancer cells and tissues [15,16], drug protection from destructive reactions, oxidization and environmental tension [17,18] and more essentially decreasing the required dosage of the anti-cancer drug in chemotherapy [19,20].

Nanovectors such as carbon nanotubes (CNT) and boron nitride nanotubes (BNNT) have attracted more attention in recent years as drug nanovehicles due to possessing remarkable features such as large surface area, very large inner volume for drug storage [21] and easy modification with desirable molecules [22,23]. Generally, nanotubes would be single-walled or multi-walled depending on the synthesis approach and subsequently different physical and chemical properties vary in terms of the number of layers [24,25]. However, due to the short-range Lenard Jones potential being mostly considered, the interaction between the outer layers of multi-walled nanotube and the drug could be negligible. Thus, the number of layers of nanotube would not obviously affect the adsorption energy of the drug inside the cavities of nanotube [26]. Several theoretical research works have been performed to predict the performance of nanotubes as cargo (drug and gene) nanocarriers. For instance, Maleki et al. [27] investigated the adsorption of the anti-cancer drug Doxorubicin on the external surface of CNT using molecular dynamics (MD) simulation. They reported that the van der Waals (vdW) interaction between the drug and the CNT determined the adsorption process due to the zero value of the electrostatic (Elec) energy. Moradnia et al. [28] simulated the adsorption of the Gemcitabine anti-cancer drug on the surface of the functionalized CNT employing the MD approach. The negative value of the free energy and vdW energy between the drug and the CNT confirmed that the drug was stably adsorbed on the nanotube. Dehneshin et al. [29] compared the adsorption kinetics of various anti-cancer drugs such as sunitinib, streptozotocin and sorafenib on the functionalized CNT by MD simulation. They reported that the adsorption of sorafenib and sunitinib on the functionalized CNT occurs through the π–π stacking and hydrogen bonding, while streptozotocin mainly participates in hydrogen bonding. The performance of the BNNT and hydroxylated BNNT as nanocarrier of the platinum drug was reported by Khatti et al. [30]. They found that the hydroxyl groups facilitate the insertion of the platinum drug into the BNNT through both hydrogen bonding and the vdW interaction between the drug and the functionalized BNNT. Roosta et al. [31] reported that the encapsulation of gemcitabine into the cavity of BNNT (18, 0) occurs spontaneously, as reflected in the negative interaction energy value of −0.9 kcal·mol^−1^.

In the light of outcomes of the previous simulations, it is well-documented that the nanotube-anticancer drug non-bonded interaction energies determining the adsorption capacity of the drug can be quite precisely computed in terms of the vdW and Elec between the nanotube and drug. MD simulation is based on the classical physics and has been widely used to simulate the biological phenomena like drug encapsulation, because of the fact that it is speedy and can additionally allow one for computation of twisting and other conformational transitions through a relatively precise manner. Thus, theoretical investigations based on MD calculations are required to shed more light on the performance of the nanotubes as nanocarriers of anti-cancer drugs, which necessitates the computation of interaction energies in the drug delivery systems.

MD simulation as a promising tool has been widely applied to investigate the performance of the systems having nanomaterials in various fields of biology and materials science [32,33]. In addition, evaluating some intrinsic properties of nanomaterials such as thermal features [34], toughness [35,36] and mechanical behaviours [37,38], this computational approach provides insight into the interatomic interactions in nanoparticle-based complex systems containing biomolecules [39]. The anti-cancer drug Fluorouracil (5-FU), with the commercial name of Adrucil^®^, is the second most common chemotherapeutic drug but causes undesired cardiotoxicity such as coronary thrombosis, coronary vasospasm and sudden cardiac death [40]. In the current work, MD simulation was applied to compare the performance of the CNT (8,8) and BNNT (8,8) as nanocarriers with the length of 20 Å for anti-cancer drug 5-FU through the calculation of vdW between the nanotube and the drug 5-FU, followed by the computing the potential of mean force (PMF) of the encapsulated drug. The storage capacity of the BNNT (8,8) with the length of 20 Å to encapsulate 5-FU molecule was also investigated.

## 2. Theoretical

**Figure 1:**
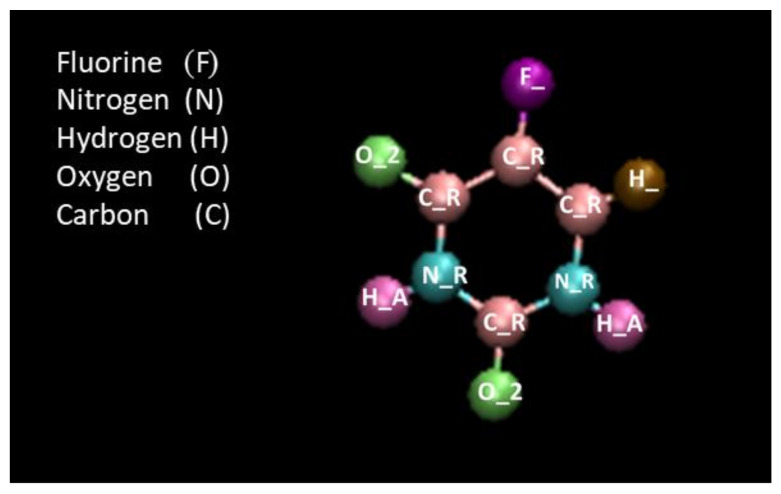
The molecular structure of the anti-cancer drug Fluorouracil (5-FU) and its atoms as represented using the DREIDING force field.

In this study, the encapsulation processes of the drug 5-FU inside the CNT and BNNT and the storage of the 5-FU inside BNNT were investigated through MD simulation by employing the Large-Scale Atomic/Molecular Simulator (LAMMPS) software. The Tersoff potential was used to consider the interaction between the atoms in the CNT and BNNT [41]. The temperature and the pressure of the systems were adjusted to 300 K and 101.3 kPa, respectively, by applying Langevin dynamics and the Langevin piston Nose–Hoover method [42]. The types of bond_style and angle_style were named harmonic. The dihedral_style and improper_style were class2 and umbrella, respectively. The non-bonded Lennard–Jones interactions were considered as short-range interaction, which was multiplied by the CHARMM switching function, described as below [43]:

(1) $$S\left(r\right)=\frac{\left[r_{out}^{2}-r^{2}\right]\left[r_{out}^{2}+2r^{2}-3r_{in}^{2}\right]}{\left[r_{out}^{2}-r_{in}^{2}\right]}$$

where $r_{in}$ and $r_{out}$ were considered 8 Å and 12 Å, respectively. Moreover, coulombic potential was considered as long-range particle-particle particle-mesh (*pppm*) solver. The parameters of Lennard-Jones potential for cross vdW interactions between non-bonded atoms were estimated using Lorentz–Berthelot combination rule [44]. The visualization was obtained using visual MD (VMD) simulation [45]. The molecular structure of the drug Fluorouracil (5-FU) is demonstrated in Figure 1.

The interaction parameters between the atoms of 5-FU were obtained from the DREIDING force field [46]. Therefore, the type of special bond was defined as DREIDING. Details on computations can be found in supplementary data. The simulation steps of this research are described as following:

(1) In the first step, the insertion of 5-FU peptide into the CNT or BBNT and subsequently, the stability of the encapsulated drug inside the nanotubes was studied. Considering the size of the 5-FU guest molecule and in order to serve as a host drug nano-carrier, the nanotube was selected due to the chirality of an armchair (8,8), having the length and diameter of 20 and 6.26 Å, respectively. At time 0 nm, the 5-FU was situated at the initial distance of 2 Å from the nanotube in the MD simulation space. The axial direction of the nanotube was set parallel to the z-axis of the simulation box. The complex comprised of the nanotube- 5FU was immersed in the simulation box consisting of TIP3P3-point water molecules and counter-ions to neutralize the simulated solution with periodic boundary conditions. To assess the encapsulation process of the peptide, in the first stage, the minimization of the system was performed in the canonical NVT ensemble at 300 K, where moles (N), volume (V) and temperature (T) gradients were conserved, while the nanotube gradients were fixed. Next, the MD runs were performed in the NPT ensemble for 15 ns with the time step of 1 fs. The vdW interaction between the drug 5-FU and the nanotube was calculated according to the below equality as [45]:
  (2) $$E_{vdW-int}\left(t\right)=E_{5FU+NT}\left(t\right)-E_{5FU}\left(t\right)-E_{NT}\left(t\right)$$
  where $E_{vdW-int}$ refers to vdW Energy between 5-FU and the capped SWCNT, $E_{5FU+NT\;}$ is vdW interaction of 5-FU combined with the capped SWCNT. $E_{5FU}$ and $E_{NT}$ stand for vdW energies of 5-FU guest and the nanotube, respectively.

To evaluate the stability of the encapsulated 5-FU inside the nanotube, an external force was loaded on the encapsulated 5-FU along the z-axis of the nanotube to pull it out from the nanotube in the direction opposite to the penetrating process. The spring constant *k* and pulling velocity were chosen equal to 15 kcal·mol^−1^ Å^−2^ and 0.005 Å ps^−1^, respectively [47]. The pulling process was simulated ten (10) times to compute the potential of PMF using Jarzynski’s equality as noted below [48]:

(3) $$e^{-\beta \Delta G}=e^{-\beta W}$$

where Δ*G* and *W* correspond to the free energy discrepancy between two states and the performed work on the system, respectively. The *β* is equal to ($K_{B}$T)^−1^, where $K_{B}$ stands for the Boltzmann constant.

(2) At the second step, the storage capacity of the BNNT (8,8) was investigated. For this, 10 molecules of 5-FU were placed inside the nanotube. The axial directions of nanotubes were set to be parallel to the z-axis of the simulation box. The minimization of the system was done in the canonical NVT ensemble at 300 K while the SWCNT was fixed. Then, the MD run was performed in the NPT ensemble for 15 ns with the time step of 1 fs.

## 3. Results and Discussion

### 3.1. Localization of Drug 5-FU within the Nanotube-Drug Complex

**Figure 2:**
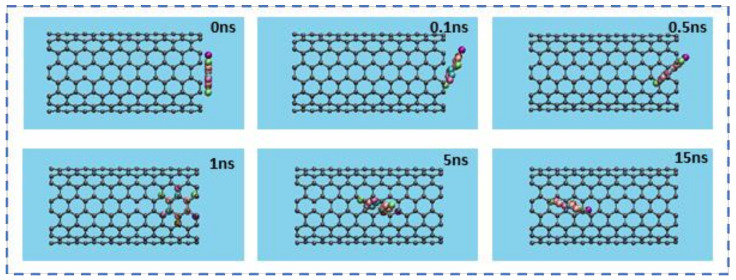
Representative snapshots of the insertion and transportation pathway of a single molecule of 5-FU into an armchair CNT (8,8) at various times. For clarity, the water molecules have been omitted from the image.

**Figure 3:**
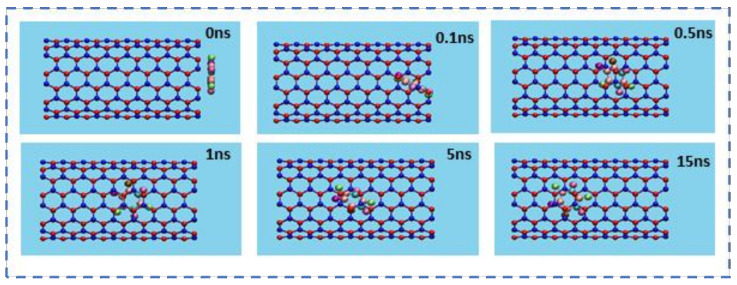
Representative snapshots of the insertion and transportation pathway of a single molecule of 5-FU into an armchair BNNT (8,8) at various times. For clarity, the water molecules have been omitted from the image.

The insertion process of the drug 5-FU inside the cavity of CNT (8,8) and BNNT (8,8) was verified through the MD simulation. The snapshots of these processes were monitored using VMD software to show the positions of the drug at various times in the simulation box. Figure 2 and Figure 3 demonstrate the snapshots of these processes for encapsulating the 5-FU inside CNT and BNNT, respectively. As can be seen, the 5-FU molecule was successfully inserted inside the cavity of nanotubes and remained stably encapsulated up to the end of the simulation at 15 ns. However, the 5-FU molecule was adsorbed into the BNNT more rapidly comparing the CNT.

**Figure 4:**
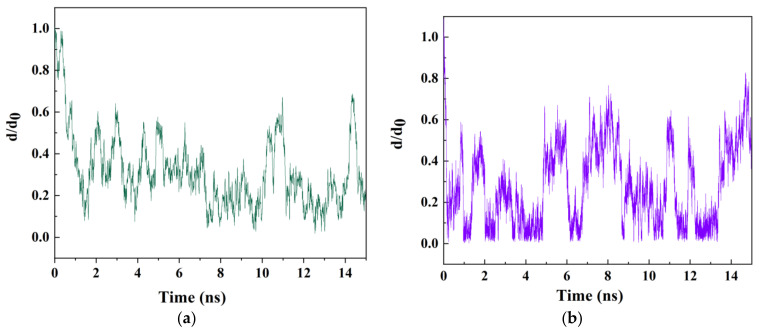
The variation of *d/d*_0_ between the drug 5-FU and CNT (8,8) (**a**) and between the drug 5-FU and BNNT (8,8) (**b**) as a function of the simulation time.

As a controlling parameter that visualizes the tendency of drug to be adsorbed onto the or keep aside from the interior walls of the nanotubes, one can consider the variation of the normalized center of mass (CoM) distance between the drug and nanotube. Right before the entrance of the drug into the nanotube, the initial CoM distance, namely *d*_0_ is equal to 12 Å. However, the CoM distance (namely, *d*) takes values less than *d*_0_, thus, the *d*/*d*_0_ (normalized CoM distance) is taken as a measure of the hydrophilicity (near to the wall) or hydrophobicity (near to the centroid of the nanotube). Figure 4a,b illustrate the variations of *d*/*d*_0_ between the drug 5-FU and CNT as well as between the 5-FU and BNNT, respectively. As expected, the *d*/*d*_0_ values for both the studied drugs, i.e., drug–CNT and drug–BNNT complexes, took very small values right in the entrance of the nanotubes (after 1.5 ns 0.1 ns of the simulation, respectively). This is a signature of rapid adsorption of the drug into the nanotubes due to the vdW interaction between the drug and nanotube. This tendency was more salient in the case of BNNT, as observed in a recent work of this group [49]. It was shown that an antimicrobial peptide was self-adjusting through conformational alterations before entrance into the BNNT. After the complete encapsulation of the drug inside both nanotubes, the values of the *d*/*d*_0_ fluctuated continuously in the specified range due to surrounding vdW interaction between the interior wall of the nanotube and the drug up to 15 ns of the simulation. 

**Figure 5:**
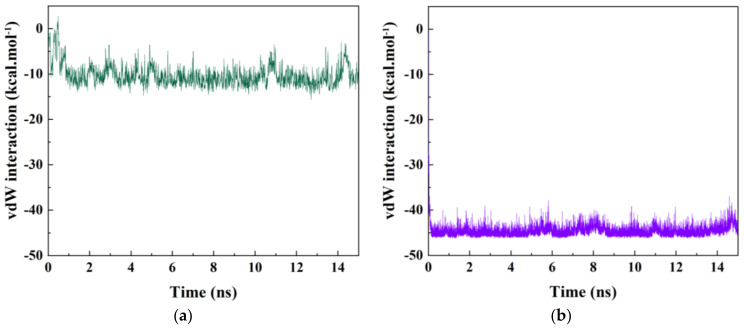
(**a**) the vdW interaction between the drug 5-FU and the CNT, (**b**) the vdW interaction between the drug 5FU and the BNNT as a function of simulation time.

The alterations in vdW interaction energy between the drug 5-FU and nanotube in the CNT-drug and BNNT-drug complexes during the encapsulation process are depicted in Figure 5a,b, respectively. As was expected, the vdW interaction energies of the CNT-drug and BNNT-drug complexes dwindled with the drop of *d*/*d*_0_ values during the encapsulation process and reached the approximate values of −10 and −45 kcal·mol^−1^, respectively after the complete insertion of the drug into the nanotube cavity at the end of the simulation process. The vdW interaction energy of the system containing BNNT decreased to a lower value, which revealed that the stronger vdW interaction between the BNNT and the drug 5FU resulted in developing a biocompatible drug delivery system with the ability to penetrate cells [50]. A similar downward trend in the vdW energy of encapsulating process was observed in the work done by Huajian et al. [51]. They reported that the DNA oligonucleotides were adsorbed into CNT through the negative value of vdW interaction energy. Hasanzade et al. [52] reported that 6-Thioguanine molecule had the strongest negative van der Waals interaction with BNNT comparing CNT, which revealed that the BNNT would be a more beneficial nanocarrier for this drug than CNT. In a previous work [49], the vdW interaction energy of the cRW3-BNNT complex dwindled during the encapsulation process reaching the value of −142.7 kcal·mol^−1^.

### 3.2. Calculation of Free Energy from the MD Simulation

**Figure 6:**
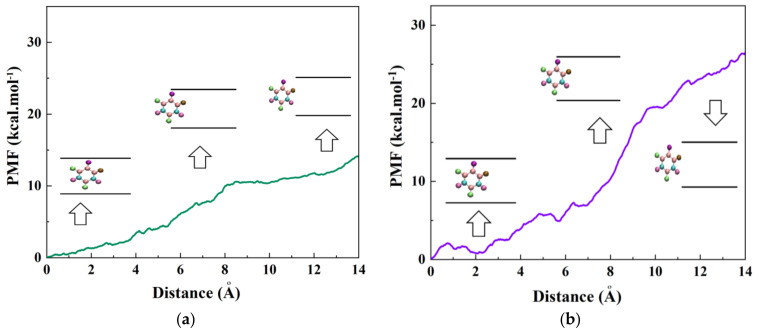
The PMF for the system comprised of a single 5-FU molecule inside (**a**) CNT (8,8), (**b**) BNNT (8,8) computed from five pullings through the MD simulation. The images represent the positions of the 5-FU molecule corresponding to the z-coordinate along the nanotube at close proximity-, entering/exiting- and in a fully enclosed position inside the nanotube.

At the end of the simulation, when the drug 5-FU was stably encapsulated inside the nanotubes at 15 ns, the PMF profiles of the adsorbed drug was computed by pulling it out through the MD simulation at the speed of 0.005 Å ps^−1^, which was chosen according to the speed of the encapsulation process. MD simulations were repeated five times by varying the initial velocity of atoms; then, the average value of the work (W) at each pulling distance was taken and shown as PMF profiles in Figure 6a,b for the systems having CNT and BNNT, respectively. Moreover, the 5-FU molecule positions along with the z-axis corresponding to the nanotubes are illustrated in these figures. As observed, the free energies of 5-FU molecules in two simulated systems containing CNT and BNNT increased during the pulling process and reached the values of 14 and 25 kcal·mol^−1^, respectively at the pulling distance of 14 Å. These results suggest that the encapsulation process of the drug inside the CNT and BNNT happened spontaneously with the free energies of −14 and −25 kcal·mol^−1^, respectively. However, the lower value of free energy for BNNT compared to the CNT is a signature of a relatively more stable encapsulation of drug inside the cavity of the BNNT. This observation is in good agreement with results obtained from the DFT and MD study done by Mortazavifar et al. [53]. They reported that the calculated adsorption energy confirmed that the drug–BNNT complex was more stable than the drug–CNT complex, which was related to the formation of the hydrogen bonding between H atoms of the drug Hydroxyurea and N atoms of the BNNT. In another work done by Veclani et al. [54], a negative value of free energy equaling to −9.5 kcal·mol^−1^ was referred to the spontaneous adsorption process of ciprofloxacin on the surface of CNT. A similar spontaneous phenomenon was reported in a recent work [55], so that the peptide HA-FD-13 was completely inserted in the cavity of BNNT with the free energy of −200.12 kcal·mol^−1^.

### 3.3. Analysis of Conformational Stability of Drug 5-FU

**Figure 7:**
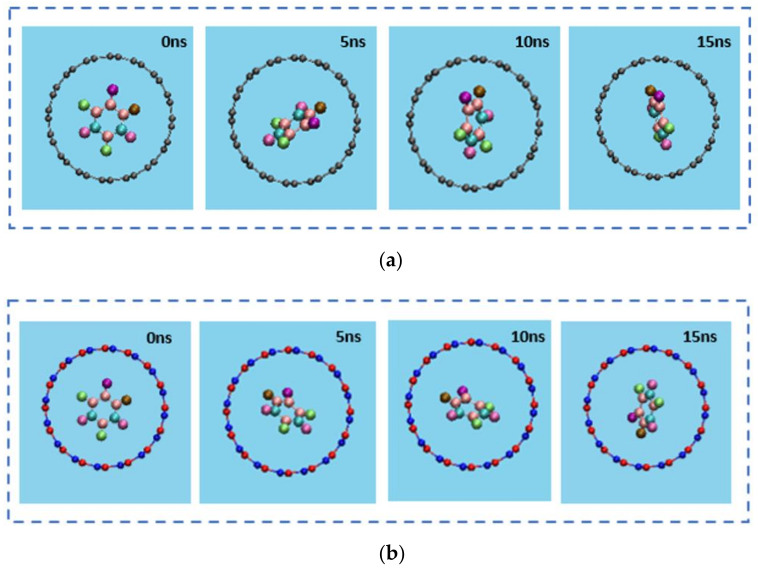
The Axial views of a single 5-FU molecule in drug–CNT complex (**a**) and in the drug–BNNT complex (**b**) from 0 ns to 15 ns, demonstrating the higher stability of 5-FU while passage through the BNNT. For clarity, the water molecules have been omitted from the image.

**Figure 8:**
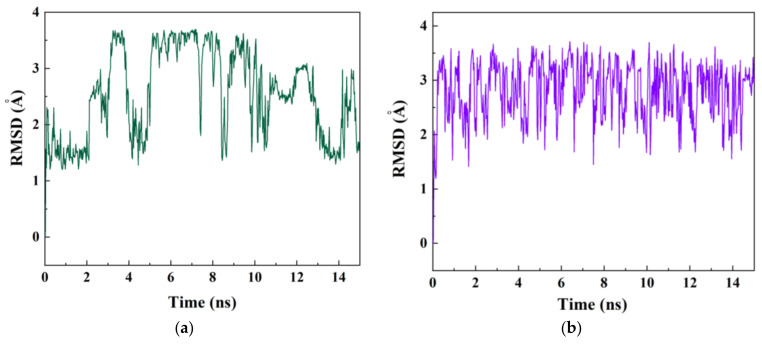

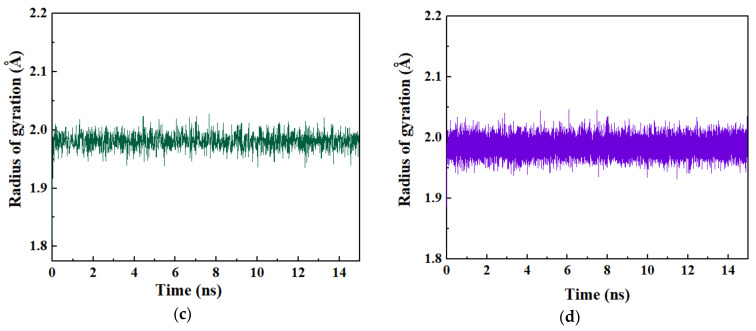
The RMSD values of 5-FU in (**a**) in the drug–CNT complex, (**b**) in the drug–BNNT complex as shown as a function of simulation time. 5-FU radius gyration(**c**) in drug–CNT complex, (**d**) in the drug–BNNT complex as a function of simulation time.

The fluctuation in the conformational changes may take place in the course of the passage of the drug 5-FU inside the nanotubes between 0 to 15 ns, which depends on the degree of hydrophobicity (adjacent to the wall) of hydrophilicity (close to the axis) of CNT and BNNT. Figure 7 compares the conformational fluctuations of 5-FU inside the CNT and BNNT based on snapshots taken over 15 ns time interval applied in MD simulation. The left-side images in both cases are representative of the drug immersed in an aqueous solution outside the nanotube, which is images theoretically right before entrance into the nanotubes, taken as 0 ns. By the time the drug was moving along the axis of nanotubes, the difference was more obvious, up to the time 15 ns, when CNT interior walls could more sensibly make sense of interaction with drug. Although drug–tube interactions in both cases have a similar nature, as featured in the localization of the drug at the vicinity of the axis, fluctuations in conformation (mainly twisting and rotation) are more salient in the case of BNNT (Figure 7b). In other words, 5-FU drug experiences a more stable stereo-conformation while passage through the BNNT (characteristic of long-standing encapsulation), rather than CNT (Figure 7a). At the end of the simulation time, the aromatic structure of the drug was adjacent to the sidewall of CNT, so that the π–π stacking interaction was induced. The root mean square deviation (RMSD) attributing to the alterations in the conformation of the 5-FU drug in CNT-drug and BNNT-drug complexes as a function of simulation time is shown in Figure 8a,b, respectively. As seen in Figure 8a, after 2 ns of the simulation, the RMSD of the drug 5-FU in a system containing CNT fluctuated continuously. However, for the system containing BNNT, the RMSD experienced continuous changes from the beginning of the simulation. This confirmed that the drug–BNNT vdW interaction was much stronger than drug–CNT vdW interaction so that the conformation of the drug was intensely under the effect of vdW interaction from the beginning of the simulation. Similar continuous variations in RMSD value of the platinum-based drug during the encapsulation inside the cavity of the silicon-carbon nanotube were observed in the MD simulation performed by Hasanzade et al. [56]. The alteration of the gyration radius of the drug in both systems containing CNT and BNT is depicted in Figure 8c,d, respectively. As observed, for both systems, the gyration radius of the drug 5-FU did not experience significant change and varied continuously surrounding the value of 2 Å due to the small size and rigid structure of the drug. However, these changes were slightly more intensive for the drug in the system having BNNT due to the stronger vdW interaction between the drug and the nanotube.

From a comparative view, the drug molecule was adsorbed into the BNNT more rapidly than into the CNT due to the stronger vdW interaction between the drug and the BNNT. According to the obtained values of the free energy of the encapsulated drug, the drug was more stable in BNNT than in the CNT, as per values of the free energy. From a clinical point of view, BNNT does not cause cell toxicity and is more biocompatible than the CNT [57]. Therefore, BNNT is more favorable candidate as a drug-carrier compared with the CNT.

### 3.4. The Storage of 5-FU inside the BNNT

**Figure 9:**
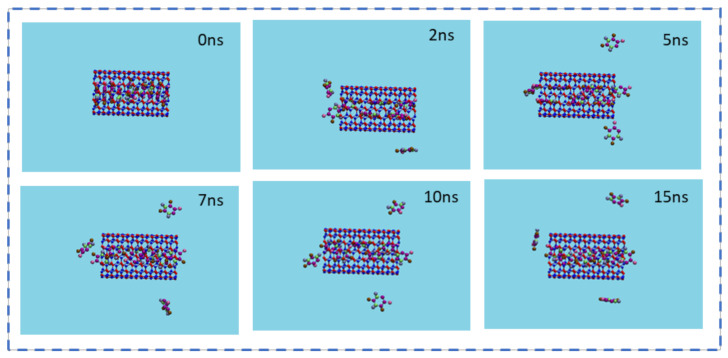
Representative snapshots of storage capacity of 5-FU molecules inside a BNNT (8,8) at various simulation times. For clarity, the water molecules have been omitted from the image.

**Figure 10:**
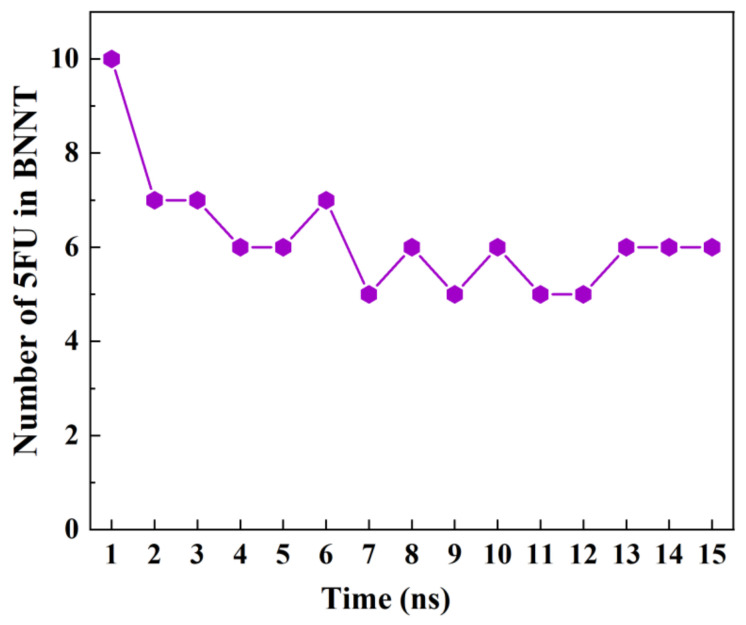
A number of 5-FU molecules enclosed inside a BNNT (8,8) as a function of simulation time.

Considering BNNT as a biocompatible and favorable nanocarrier, computing its storage capacity brings a deeper insight into the required drug amount for developing a nano-drug delivery system having the highest efficiency. Figure 9 illustrates the snapshots of the simulation of the storage of several drugs 5-FU in BNNT (8,8) at various times. Figure 10 shows the number of drugs encapsulated in BNNT as a function of simulation time. At 0 ns of the simulation, teen numbers of the drugs were placed in the cavity of the BNNT. As the simulation initiated, some of the drugs exited the cavity and presented surrounding the exterior wall of the nanotube due to the vdW interactions. It was observed that the average six numbers of the drugs would be stably encapsulated inside the BNNT.

## 4. Conclusions

In the current work, the encapsulation process of the anti-cancer drug 5-FU inside the BNNT and CNT was studied, followed by calculation of the numbers of the drugs that can be encapsulated in the BNNT cavity through the molecular dynamics (MD) simulation. Regarding the encapsulation process of one drug 5-FU in the cavity of nanotubes, it was observed that the drug was rapidly adsorbed inside the nanotubes and remained stably encapsulated up to the end of the simulation. However, this adsorption process was accelerated for the drug–BNNT complex due to the stronger van der Waals (vdW) interaction energy between the drug and BNNT. The values of vdW interaction energy in systems containing CNT and BNNT decreased to the values of the −15 and −45 kcal·mol^−1^, respectively at the end of the simulation (15 ns), which was in favor of the adsorption process. Free energy calculations revealed that the encapsulation process of the drug inside the CNT and BNNT occurred spontaneously with the free energies of −14 and −25 kcal·mol^−1^, respectively. However, the lower value of the free energy in the system containing the BNNT revealed more stability of the encapsulated drug inside the cavity of the BNNT comparing the system having CNT. In terms of the storage capacity of the BNNT, it was clarified that the average six numbers of the drugs would be stably encapsulated inside the BNNT cavity.

## Data Availability

All data presented in this manuscript as well as complementary information will be available upon request from Amin Hamed Mashhadzadeh (A.H.M.); amin.hamed.m@gmail.com & amin.hamedmashhadzadeh@nu.edu.kz.

## Supplementary Materials

The following are available online. Details on Computations: Table S1. Van der Waals parameters of 5-FU atoms, Table S2. $K_{e}$ and $R_{e}$ constants for bond types of drug 5-FU, Table S3. Quantities of $\theta_{J}^{{}^{\circ}}$ and $K_{IJK}$ for angle types of the drug 5-FU, Table S4. The values of $V_{\mathrm{JK}}$, $n_{IL}$, and $\varphi_{JK}^{{}^{\circ}}$ for dihedral types of the drug 5-FU, Table S5. Values of $K_{I}$ for improper types of the drug 5-FU, Table S6. Atomic charges of elements in drug 5-FU.

## Sample Availability

Samples of outcomes are available as upon request from Amin Hamed Mashhadzadeh (A.H.M.); amin.hamed.m@gmail.com & amin.hamedmashhadzadeh@nu.edu.kz.
