# Supplementary material for: Theoretical Encapsulation of Fluorouracil (5-FU) Anti-Cancer Chemotherapy Drug into Carbon Nanotubes (CNT) and Boron Nitride Nanotubes (BNNT)
Source: Molecules. 2021 Aug 13;26(16):4920. doi: 10.3390/molecules26164920 (PMC8398462; doi:10.3390/molecules26164920)
Supplement: Supplementary file 1 [file molecules-26-04920-s001.zip › molecules-1307310-supplementary.pdf]

Supplementary information to:

Theoretical encapsulation of Fluorouracil (5-FU) anti-cancer chemotherapy drug into carbon nanotubes (CNT) and boron nitride nanotubes (BNNT)

Maryam Zarghami Dehaghani[1], Farrokh Yousefi[2], S. Mohammad Sajadi[3,4], Muhammad Tajammal Munir[3], Otman Abida[5], Sajjad Habibzadeh[6,*], Amin Hamed Mashhadzadeh[7,*], Navid Rabiee[8], Ebrahim Mostafavi[9,10,*], Mohammad Reza Saeb[11]

[1] *Center of Excellence in Electrochemistry, School of Chemistry, College of Science, University of Tehran, Tehran, Iran*

[2] *Department of Physics, University of Zanjan, 45195-313, Zanjan, Iran*

[3] *Department of Nutrition, Cihan University-Erbil, Kurdistan Region, Iraq*
[4] *Department of Phytochemistry, SRC, Soran University, KRG, Iraq*

[5] *College of Engineering and Technology, American University of the Middle East, Kuwait*

[6] *Department of Chemical Engineering, Amirkabir University of Technology (Tehran Polytechnic), Tehran, Iran*

[7] *Mechanical and Aerospace Engineering, School of Engineering and Digital Sciences, Nazarbayev University, Nur-Sultan 010000, Kazakhstan*

[8] *Department of Chemistry, Sharif University of Technology, Tehran, Iran*

[9] *Stanford Cardiovascular Institute, Stanford University School of Medicine, Stanford, CA, USA*

[10] *Department of Medicine, Stanford University School of Medicine, Stanford, CA, USA*

[11] *Department of Polymer Technology, Faculty of Chemistry, Gdańsk University of Technology, G. Narutowicza 11/12 80-233, Gdańsk, Poland*

* Correspondence: Ebrahim Mostafavi, ebimsv@stanford.edu; Amin Hamed Mashhadzadeh, amin.hamed.m@gmail.com ; Sajjad Habinzadeh, sajadhabibzadeh@gmail.com

Details on Computations

The potential energy of the molecule 5-FU (E) is defined as below [1]:

$$E = E_{val} + E_{nb} \quad \text{(S1)}$$

where E_{val} is valence or bonded interactions, and E_{nb} refers to non-bonded interactions which are the superposition of the van der Waals energy (E_{vdW}) and Coulombic energy (E_Q) (as shown in Eq. S2) [1]:

$$E_{nb} = E_{vdW} + E_Q \quad \text{(S2)}$$

E_{vdW} depends on the two parameters of D_0 and R_0 , which are van der Waals well depth (kcal.mol^{-1}), and van der Waals bond length (as defined in Eq. S3) [1]. The obtained values of the D_0 and R_0 for each atom type of the drug 5-FU are tabulated in Table S1.

$$E_{vdW} = D_0((\frac{R}{R_0})^{-12} - 2(\frac{R}{R_0})^{-6}) \quad \text{(S3)}$$

Table S1. Van der Waals parameters of 5-FU atoms.

**Atom Type**	**R_0 (Å)**	**D_0 (Kcal.mol^{-1})**
C_R	3.458	0.0950
N_R	3.293	0.1450
F_	2.928	0.3050
O_2	3.128	0.2150
H___A	2.852	0.0100
H_	2.852	0.0100

The valence interaction comprises of the bonding stretch (E_B), bond-angle bend (E_A), dihedral angle torsion (E_T), and inversion terms (E_I) as represented in Eq. S4 [1]:

$$E_{val} = E_B + E_A + E_T + E_I \quad \text{(S4)}$$

The Bond stretch interaction is described as below [1]:

$$E_B = K_e(R - R_e)^2 \quad \text{(S5)}$$

The force constant (K_e) and anharmonic terms near equilibrium (R_e) for bond types of the drug 5-FU are reported in Table S2.

Table S2. K_e and R_e constants for bond types of drug 5-FU

Bond Type	K_e (Kcal.mol^{-1}. Å^{-2})	R_e (Å)
C_R-N_R	350	1.340
C_R-C_R	525	1.390
C_R-O_2	700	0.970
N_R-H___A	350	1.020
C_R-H_	350	1.020
C_R-F_	350	1.301

The bond-angle bend interaction energy (E_A) between is defined in Eq. S6 [1]. The values of equilibrium angle ($\mathring{\theta_J}$) and force constant (K_{IJK}) for angle types of the drug 5-FU are summarized in Table S3.

$$E_A = E_{IJK} = K_{IJK}(\theta_{IJK} - \mathring{\theta_J})^2 \tag{S6}$$

Table S3. Quantities of $\mathring{\theta_J}$ and K_{IJK} for angle types of the drug 5-FU

Angle Type	K_{IJK} (kcal.mol^{-1}.rad^{-2})	$\mathring{\theta_J}$ (rad)
C_R-C_R-N_R	50	120.00
N_R-C_R-O_2	50	120.00
C_R-C_R-O_2	50	120.00
C_R-N_R-C_R	50	120.00
C_R-N_R-H___A	50	120.00
N_R-C_R-N_R	50	120.00
N_R-C_R-H_	50	120.00
C_R-C_R-H_	50	120.00
C_R-C_R-C_R	50	120.00
C_R-C_R-F_	50	120.00

The torsion or dihedral interaction energy (E_D) for two bonds IJ and KL connected with bond is defined as below [1]:

$$E_D = E_{IJKL} = V_{JK}(1 - \cos(n_{IL}(\varphi - \mathring{\varphi}_{JK}))) \tag{S7}$$

where V_{JK}, n_{IL}, and $\mathring{\varphi}_{JK}$ correspond to the barrier to rotation, the periodicity, and the equilibrium angle constants. The values of these constants for dihedral types of the drug 5-FU are summarized in Table S4.

Table S4. The values of V_{JK}, n_{IL}, and φ°_{JK} for dihedral types of the drug 5-FU

Dihedral Type	V_{JK} (Kcal.mol^{-1})	n_{IL}	φ°_{JK}
C_R-C_R-N_R-C_R	5	2	0
C_R-C_R-N_R-H___A	5	2	0
O_2-C_R-N_R-C_R	5	2	0
O_2-C_R-N_R-H___A	5	2	0
C_R-C_R-C_R-N_R	5	2	0
N_R-C_R-C_R-F_	5	2	0
C_R-C_R-C_R-O_2	5	2	0
F_-C_R-C_R-O_2	5	2	0
N_R-C_R-N_R-C_R	5	2	0
N_R-C_R-N_R-H___A	5	2	0
H_-C_R-N_R-C_R	5	2	0
H_-C_R-N_R-H___A	5	2	0
C_R-C_R-C_R-H_	5	2	0
F_-C_R-C_R-H_	5	2	0

The improper (inversion) interaction energy (E_I) for drug 5-FU with planar equilibrium geometry is considered in Eq. S8 [1].

$$E_I = E_{IJKL} = K_I(1 - cos\Psi_I) \qquad \text{(S8)}$$

where Ψ_I is an angle between the IL bond and JIK plane. The values of force constant K_I for improper types of the drug 5-FU are listed in Table S5.

Table S5. values of K_I for improper types of the drug 5-FU

Improper Type	K_I (kcal.mol-^{1}.rad^{-2})
C_R-C_R-N_R-O_2	40.00
C_R-N_R-C_R-H___A	40.00
N_R-C_R-N_R-O_2	40.00
C_R-C_R-N_R-H_	40.00
C_R-C_R-C_R-F_	40.00

The partial charges of atoms in drug 5-FU are reported in Table S6.

Table S6. Atomic charges of elements in drug 5-FU

Atom	Charge
C_R	0.495100
N_R	-0.494500
C_R	0.590000
N_R	-0.526200
C_R	-0.001900
C_R	0.487600
F_	-0.550500
O_2	-0.390100

O_2	-0.370200
H___A	0.289100
H___A	0.287200
H_	0.184300

Reference

1. Mayo, S.L., B.D. Olafson, and W.A. Goddard, *DREIDING: a generic force field for molecular simulations.* Journal of Physical chemistry, 1990. **94**(26): p. 8897-8909.
